# Comparative analysis of the human serine hydrolase OVCA2 to the model serine hydrolase homolog FSH1 from *S*. *cerevisiae*

**DOI:** 10.1371/journal.pone.0230166

**Published:** 2020-03-17

**Authors:** Jessica S. Bun, Michael D. Slack, Daniel E. Schemenauer, R. Jeremy Johnson

**Affiliations:** Department of Chemistry and Biochemistry, Butler University, Indianapolis, Indiana, United States of America; Karl-Franzens-Universitat Graz, AUSTRIA

## Abstract

Over 100 metabolic serine hydrolases are present in humans with confirmed functions in metabolism, immune response, and neurotransmission. Among potentially clinically-relevant but uncharacterized human serine hydrolases is OVCA2, a serine hydrolase that has been linked with a variety of cancer-related processes. Herein, we developed a heterologous expression system for OVCA2 and determined the comprehensive substrate specificity of OVCA2 against two ester substrate libraries. Based on this analysis, OVCA2 was confirmed as a serine hydrolase with a strong preference for long-chain alkyl ester substrates (>10-carbons) and high selectivity against a variety of short, branched, and substituted esters. Substitutional analysis was used to identify the catalytic residues of OVCA2 with a Ser117-His206-Asp179 classic catalytic triad. Comparison of the substrate specificity of OVCA2 to the model homologue FSH1 from *Saccharomyces cerevisiae* illustrated the tighter substrate selectivity of OVCA2, but their overlapping substrate preference for extended straight-chain alkyl esters. Conformation of the overlapping biochemical properties of OVCA2 and FSH1 was used to model structural information about OVCA2. Together our analysis provides detailed substrate specificity information about a previously, uncharacterized human serine hydrolase and begins to define the biological properties of OVCA2.

## Introduction

Serine hydrolases are a large, ubiquitous family of enzymes grouped based on their ability to perform hydrolysis reactions on a range of biological substrates.[1–3] Over 200 serine hydrolases have been identified in humans with their function split near equally between serine proteases, which hydrolyze amide bonds in peptides and proteins, and metabolic serine hydrolases, which hydrolyze ester, thioester, amide, and epoxide bonds in small molecules, peptides, or proteins.[3] With their diverse substrates, metabolic serine hydrolases have roles in a wide range of biological processes from standard functions, including metabolism, immune response, pain sensation, and neurotransmission, to disease related conditions, such as cancer and bacterial infection.[1, 3–5]

The diverse, essential functions performed by metabolic serine hydrolases have also made them therapeutic targets.[1] Drugs targeting serine hydrolases have been approved for the treatment of obesity, diabetes, and dementia with development of new serine hydrolase inhibitors targeting pain, cancer, and high triglycerides.[1, 6–8] Methodological advancements such as activity based protein profiling (ABPP) and especially ABPP combined with unbiased inhibitor screens have assigned biological substrates, functions, and pathways to previously uncharacterized metabolic serine hydrolases.[4, 9–12] Yet, many members of the 100 plus human metabolic serine hydrolase family remain uncharacterized, including members with preliminary evidence indicating that they may be clinically relevant.[3]

Among these potentially clinically-relevant but uncharacterized human serine hydrolases is OVCA2, a serine hydrolase originally named Ovarian Tumor Suppressor Candidate 2 based on its genetic association with tumorigenesis and a region of human chromosome 17 that is deleted in 80% of cancer.[3, 13] Although the other locus (OVCA1) within this region of chromosome 17 has been tracked as the tumor suppressor,[14, 15] OVCA2 has still been linked with a variety of cancer-related processes, including being upregulated in four cancer cell lines, interacting with central apoptosis signals, and differential expression with anticancer agents.[16, 17] Recently an expansive CRISPR screen also pinpointed OVCA2 as a protective factor for acetaldehyde toxicity, a metabolite of ethanol processing.[18] Disruption of the *OVCA2* gene led to increased acetaldehyde sensitivity and accumulation of the DNA adduct, *N*^2^-ethylidene-dG, a by-product of acetaldehyde buildup.

**Fig 1:**
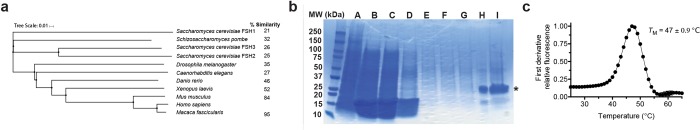
Expression and isolation of OVCA2. (A) Phylogenetic relationship between OVCA2 and homologous serine hydrolases across various model organisms. The amino acid sequence of OVCA2 was aligned with the 10 homologues and a cladogram of the aligned proteins was constructed with percent similarities from Clustal Omega. Detailed sequence analysis is given in S1 Table. (B) Purification of OVCA2. OVCA2 was purified according to the procedure outlined in the Experimental Procedures. An SDS–PAGE gel (4–20%) showing the protein purification of OVCA2 with samples from different stages in the purification: pellet after lysis (a), supernatant after lysis (b), flowthrough after 10 mM washes imidazole (c and d), 25 mM washes imidazole (e and f), 50 mM wash imidazole (g), and 250 mM elution imidazole (h), and final protein sample after dialysis (i). The expected molecular weight of our N-terminal tagged version of OVCA2 is 27.0 kDa. The molecular weight was confirmed by comparison to the Kaleidoscope prestained protein standard (Bio-Rad). (C) Thermal stability of OVCA2. The folded to unfolded transition for wild-type OVCA2 (0.3 mg/mL in PBS) was observed by DSF. The measurement was completed in triplicate and is shown ± SD. The majority of the error is smaller than the size of the data marker.

Detailed sequence analysis of OVCA2 places it firmly into the metabolic serine hydrolase superfamily with homologues across the kingdoms of life from fungi to plants to humans (Fig 1 and S1 Table).[3, 17] Under standard conditions, OVCA2 mRNA and protein are ubiquitously expressed and OVCA2 protein is localized across the cytoplasm and nucleoplasm, suggesting a general biological function.[19, 20] Protein interaction maps provide limited interaction partners and include only general cell organization proteins, like myosin (Myh11), desmoplakin (Dsp), and phosphatidyl glycan anchor (Pigs).[21]

Basic biological properties of OVCA2 have also been inferred based on analogy to three distant homologues from *Saccharomyces cerevisiae*.[22] These three homologous serine hydrolases were initially identified in a combined ABPP and bioinformatics screen of serine hydrolases in *S*. *cerevisiae* and were named FSH1, FSH2, and FSH3 for Family of Serine Hydrolases.[22] Structural analysis of FSH1 confirmed the FSH family as serine hydrolases with a classic catalytic triad of Ser-His-Asp and an α/β hydrolase protein fold.[23] Labeling of the catalytic serine with a covalent inhibitor in the crystal structure of FSH1 allowed conclusive assignment of the nucleophilic serine and hydrophobic binding pocket.[23] The biological functions of the three FSH *S*. *cerevisiae* hydrolases are however unknown with limited support for specific functions in *S*. *cerevisiae* outside of regulation of FSH3 expression by the Crt1 DNA damage pathway and of global assignment of their localization to the cytoplasm and nucleus.[24–26] For OVCA2, global ABPP screens have also identified it as active against hydrophobic, covalent activity based ligands, but no further analysis has been performed.[22, 27, 28]

Herein, we developed a heterologous expression and purification system for OVCA2 and used the resulting well-folded protein to characterize the detailed biochemical properties of OVCA2. Specifically, we measured the comprehensive enzymatic activity and substrate specificity of OVCA2 against libraries of small molecule fluorogenic esters and longer chain chromogenic esters. Through sequence analysis and mutagenesis, we assigned the catalytic triad of OVCA2 and confirmed its classification as a serine hydrolase. Finally, we compared the relative biochemical properties of human OVCA2 to the *S*. *cerevisiae* homologue FSH1 to determine if FSH1 is a valid structural and biochemical analog for OVCA2. Using this comparative analysis of OVCA2 and FSH1, we describe the conservation of structural and enzymatic features across this metabolic serine hydrolase family.

## Results and discussion

### Expression and isolation of OVCA2

OVCA2 is a highly conserved serine hydrolase with homologues across kingdoms of life from fungi to plants to humans (Fig 1A). Human OVCA2 shows a high degree of similarity to a single protein in many multicellular organisms, with greater than 30% similarity to homologues from *D*. *melanogaster* to *M*. *musculus* (Fig 1A and S1 Table). This similarity decreases with fungal homologues with only 21–26% overlap with the *S*. *cerevisiae* FSH proteins. FSH1, the proposed structural model for OVCA2, has 21% similarity and is the closest sequence homolog found in the PDB.[23]

Expression and isolation of pure, active OVCA2 protein proved difficult in *E*. *coli*. Shifts in expression conditions (temperature for growth and induction; induction point based on growth phase), expression strains (standard expression strains to strains containing co-chaperone expression), and expression vectors (attachment of varying purification tags and solubilization agents) failed to yield high quantities of active OVCA2. OVCA2 contains two cysteine residues that were within disulfide bonding distance in the modeled structure of OVCA2 and we hypothesized that this disulfide bridge might be important to the folding and stability of OVCA2.[17] Using a combination of the Origami B (DE3) pLyS *E*. *coli* expression strain that facilitates disulfide bond formation in *E*. *coli*, the EnPresso expression system that utilizes optimized sugar release to extend the exponential growth phase, and lowered IPTG levels for slower induction, we were finally able to obtain sufficient quantities of pure OVCA2 protein (Fig 1B) for our detailed analysis.[29]

OVCA2 was expressed with an N-terminal 6x histag and purified by Ni-metal affinity chromatography to greater than 95% purity (Fig 1B). Incubation of purified OVCA2 with 5,5′‐dithiobis(2‐nitrobenzoic acid) (Nbs_2_), which detects free thiol groups, yielded negligible levels of detectable free thiols (0.21 ± 0.02) in OVCA2, indicating that expression of OVCA2 in the Origami B (DE3) pLyS *E*. *coli* cell line led to the formation of disulfide bonds between any surface exposed cysteine residues.[30–32] Thermal stability measurements confirmed that the purified OVCA2 protein was stable at room temperature (*T*_M_ = 47 ± 0.9°C) and showed a clear folded to unfolded transition (Fig 1C). Obtaining pure, well-folded OVCA2 protein allowed us to investigate the detailed biochemical and enzymatic properties of OVCA2.

### Comprehensive substrate specificity of OVCA2

**Fig 2:**
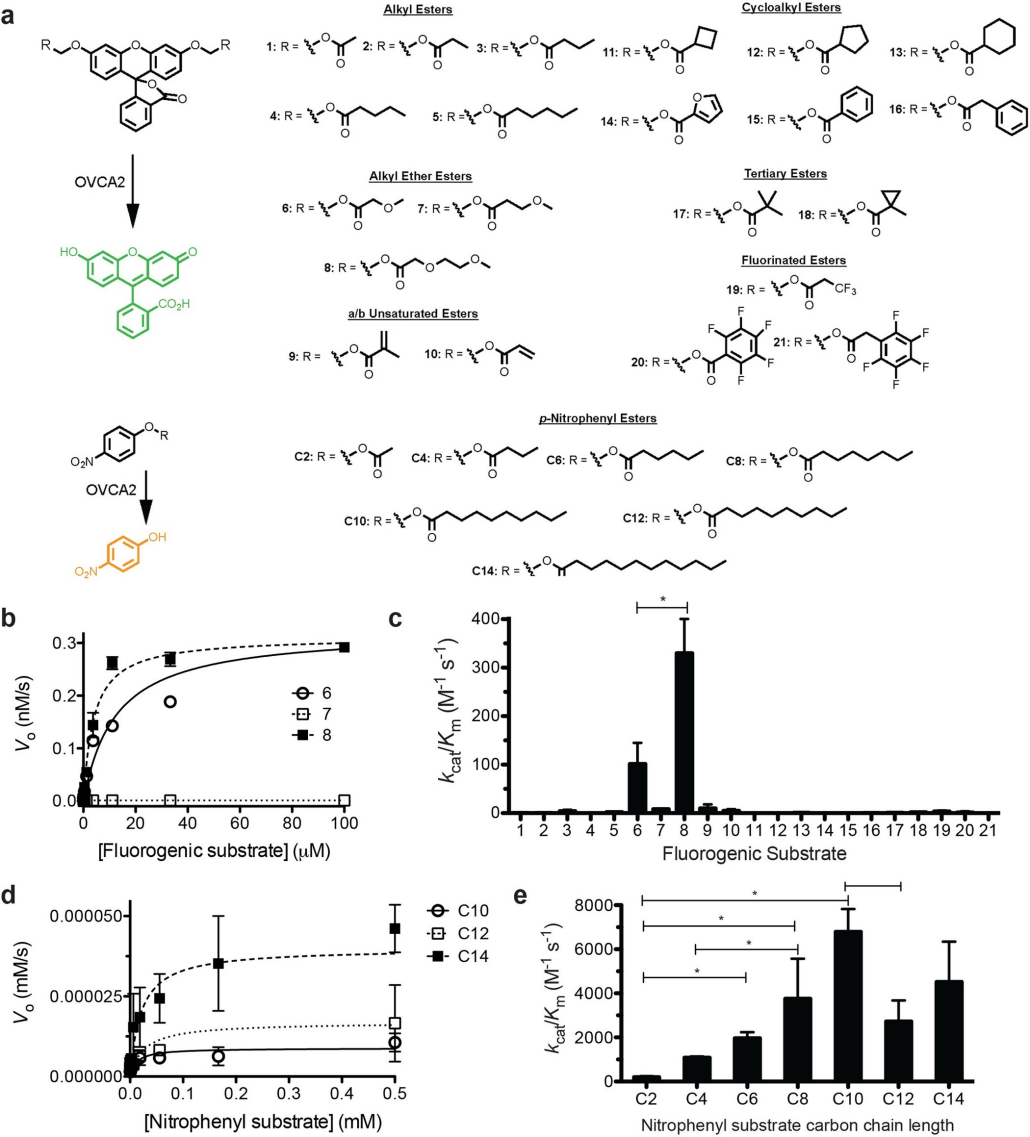
Substrate specificity of OVCA2 against two ester substrate libraries. (A) Two substrate libraries. For fluorogenic substrates, hydrolysis of the ester bond on the diacyloxymethyl ether fluorescein substrates by OVCA2 converts the fluorescein core from the nonfluorescent lactone form to the highly fluorescent quinoid form. Each of the substrates is composed of diacyloxymethyl ether fluorescein (1A) with varying R-groups. The differing R-groups have been organized into classes based on chemical functionality. All of the substrates were synthesized as described previously [35, 36, 40]. For chromogenic substrates, hydrolysis of the ester bond on the *p*-nitrophenyl substrates liberates *p-*nitrophenol, releasing its bright yellow color. Chromogenic substrates differ only in the length of the alkyl ester chain from the two-carbon *p-*nitrophenyl acetate to the 14-carbon *p-*nitrophenyl myristate. (B) Kinetic activity of OVCA2 against substrates **6** (open circles), **7** (open squares), and **8** (closed squares). All measurements completed in triplicate and shown ± SD. (C) Global comparison of the catalytic specificity (*k*_cat_/*K*_M_) of OVCA2 against each of the 21 substrates (structures and numbering given in Fig 2A). Kinetic values for substrates **6** and **8** were significantly different at *p* < 0.05. All other *k*_cat_/*K*_M_ values were significantly different from both substrates **6** and **8** at *p* < 0.05 (Unpaired two-tailed t-test; GraphPad Prism 5). (D) Kinetic activity of OVCA2 against *p-*nitrophenyl decanoate (C10), *p-*nitrophenyl laurate (C12), and *p-*nitrophenyl myristate (C14). Data points were fitted to the Michaelis-Menten equation and are shown ± SD. (E) Catalytic efficiency of OVCA2 against *p*-nitrophenyl substrates. Catalytic efficiency values (*k*_cat_/*K*_m_) are given ± SD. Values designated with an asterisk (*) were significantly different from each other at *p* < 0.05 (Unpaired two-tailed t-test; GraphPad Prism 5). For clarity, not all significant differences are shown. Labeling is instead focused on the central patterns for substrate specificity comparisons. Detailed kinetic results for all kinetic data are provided in S2 Table.

OVCA2 was previously labeled by a nonspecific diisopropyl fluorophosphonate probe and by the pan-specific serine hydrolase inhibitor methyl-arachidonyl fluorophosphonate (MSFP).[22, 27] This labelling showed that OVCA2 is a serine hydrolase with a reactive serine nucleophile, but as these probes are designed to label serine hydrolases with a wide-range of biological substrates, this reactivity did not differentiate the substrate specificity of OVCA2 from other metabolic serine hydrolases.[11, 33] To comprehensively characterize the substrate specificity of OVCA2, we utilized two libraries of ester substrates (Fig 2A). The first library was composed of fluorogenic substrates whose semi-immolative ester linkages mask the bright fluorescence of fluorescein (Fig 2A).[34] These fluorogenic substrates provide low background hydrolysis, sensitive kinetic measurements, and a broad screen of ester substrates across subclasses of metabolic serine hydrolases.[35–38] In addition to these substrates, chromogenic *p*-nitrophenyl ester substrates with varying alkyl chain lengths from 2- to 14-carbons were used to subclassify OVCA2 based on substrate preference for more lipophilic substrates.[36, 39] These *p*-nitrophenyl ester substrates have greater solubility and only one ester group for chromogenic protection, but have a higher background hydrolysis rate and lower sensitivity than the fluorogenic substrates.[34, 37]

OVCA2 showed classic Michaelis-Menten kinetics with each of these substrate classes (Fig 2B and 2D). From plots relating initial velocity and substrate concentration, values for kinetic constants (*k*_cat_, *K*_M,_ *k*_cat_/*K*_M_) were calculated and compared. Based on catalytic efficiency (*k*_cat_/*K*_M_), OVCA2 showed weak activity (<350 M^-1^ s^-1^) toward all 21 fluorogenic substrates with highest activity toward extended alkyl ether ester substrates (**6** and **8**; Fig 1C and S2 Table), which have been the highest activity substrates toward a range of enzymes.[40–43]

This low activity toward small ester substrates was also reflected in activity measurements toward the chromogenic *p*-nitrophenyl ester substrates with weak activity toward the shortest acetyl (C2) and butyl (C4) ester substrates (Fig 2E and S2 Table). In comparison, OVCA2 showed robust activity toward longer ester substrates of >8-carbons with similar catalytic efficiency toward substrates of 8- to 14-carbons (Fig 2D and S2 Table). This substrate profile, with high selectivity for extended hydrophobic alkyl esters but against shorter, branched, or bulky esters, places OVCA2 closer to the lipase subclass of serine hydrolases.[39, 44]

A narrow, extended hydrophobic binding pocket was previously modeled for OVCA2.[17] This modeled binding pocket had sufficient space to accommodate the preferred straight chain 8- to 14-carbon *p*-nitrophenyl alkyl esters and could select against the smaller fluorogenic esters. The modeled structures of OVCA2 and its *S*. *cerevisiae* homologue FSH1 did not however contain a lid domain present in classic lipases.[17, 45–47] Instead, the structure of FSH1 had a small cap domain that did not undergo large scale rearrangement upon substrate binding.[23] Overall, the comprehensive enzymatic characterization of OVCA2 shows that it is an active metabolic serine hydrolase with high selectivity against short ester substrates, but high activity toward extended alkyl chain esters.

### Assignment of catalytic amino acids

**Fig 3:**
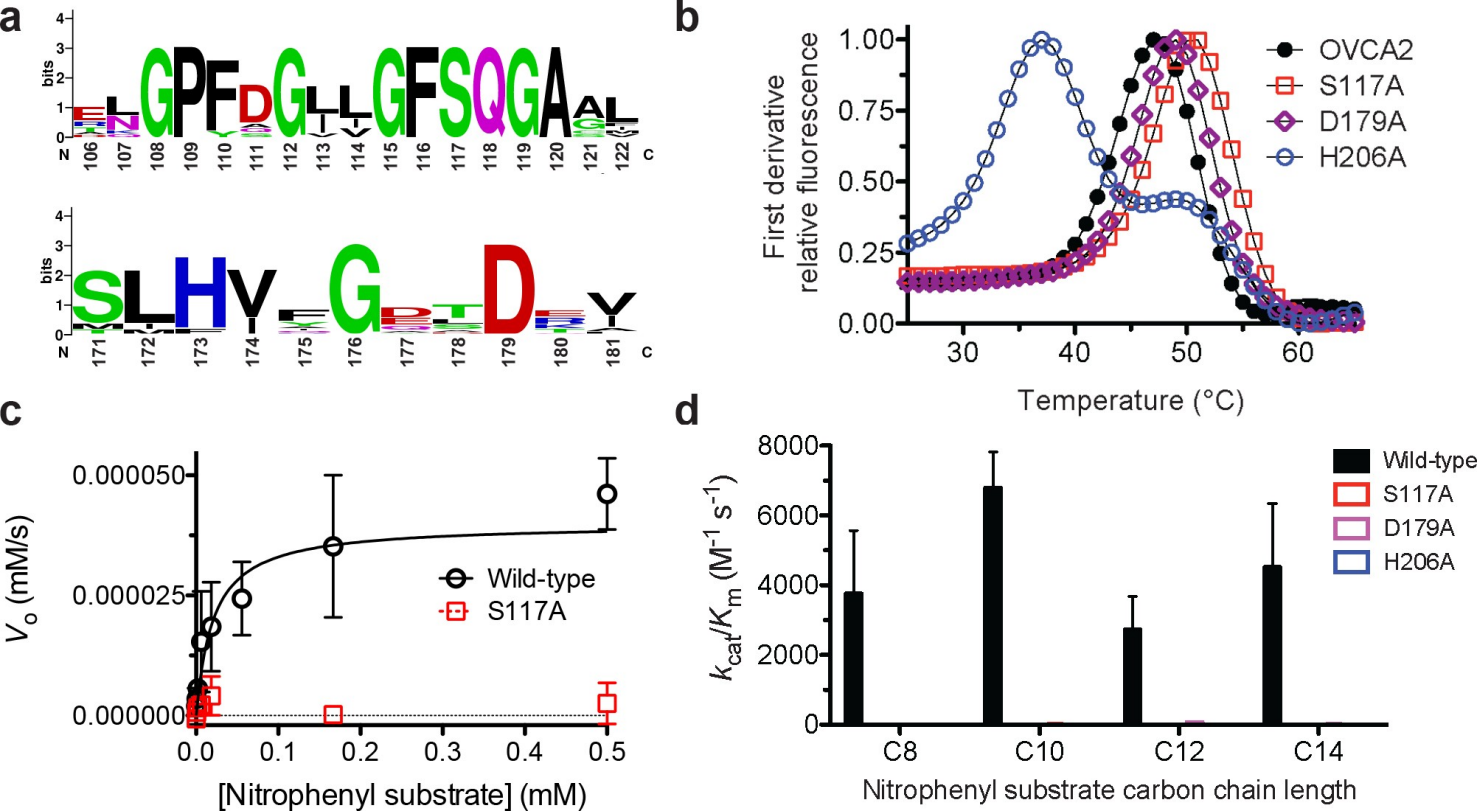
Identification of the catalytic amino acids for OVCA2. (A) Sequence conservation of residues adjacent to the proposed catalytic triad. Relative weightings and motif analysis performed using Weblogo [62]. Detailed sequence analysis given in S1 Table. (B) Thermal stability of OVCA2 variants. The thermal stability of each variant was determined by DSF. The measurement was completed in triplicate and is shown ± SD. (C) Kinetic activity of OVCA2 variants. The kinetic activity of wild-type OVCA2 (closed black circles) and the S117A variant (open red squares) were determined against the highest activity substrate, *p*-nitrophenyl decanoate (C10). All measurements were completed in triplicate and are shown ± SD. Data were fitted to the Michaelis-Menten equation using Graphpad Prism 5.0. (D) Relative catalytic activity of OVCA2 variants. The catalytic activity of each of the OVCA2 variants was determined against *p*-nitrophenyl substrates of 8- to 14-carbons. All *k*_cat_/*K*_M_ values were significantly different from wild-type OVCA2 at *p* < 0.05 (Unpaired two-tailed t-test; GraphPad Prism 5). Detailed kinetic and thermal stability analysis for OVCA2 variants are given in S4 Table with comparison of OVCA2 variant and background hydrolysis rates in S3 Fig.

Based on the robust enzymatic activity of OVCA2 to a range of long-chain alkyl esters, OVCA2 can be conclusively assigned as a serine hydrolase (Fig 2). Within serine hydrolases, multiple catalytic arrangements exist for performing the hydrolysis reaction from the classic Ser-His-Asp catalytic triad to the less common catalytic triad of Ser-Ser-Lys and catalytic diads of Ser-Lys or Ser-Asp.[2, 3] Using a combination of sequence alignment and mutagenesis, we wanted to confirm the catalytic arrangement of OVCA2. Based on sequence alignment (S1 Table), we hypothesized that the catalytic triad of OVCA2 was composed of Ser117, Asp179, and His206. The nucleophilic serine (Ser117) was found within the G-x-S-x-G motif that demarcates the nucleophilic elbow and facilitates the proper angle of the serine for catalysis.[2, 23] The sequence of the intervening “x” residues in the G-x-S-x-G motif can be used to subdivide serine hydrolase superfamilies,[48, 49] so the complete conservation of the G-F-S-Q-G motif across OVCA2 homologues confirms their high degree of homology (Fig 3A). The catalytic aspartate (Asp179) and histidine (His206) were then identified based on their complete conservation in the sequence alignment and by analogy to the structure of FSH1 (Fig 3A).[23]

Each of these residues was substituted individually with alanine and the resulting OVCA2 variants purified to homogeneity and examined for their folding and catalytic activity (Fig 3). As seen previously,[41, 42] release of the strain on the catalytic serine by substitution with alanine (S117A) slightly increased the overall stability of OVCA2 (Δ*T*_M_ = + 3°C) as did removal of the buried charged aspartate (D179A Δ*T*_M_ = + 2°C) (Fig 3B). In comparison, substitution of the catalytic histidine, which is normally buried within the α/β hydrolase protein fold,[2, 40] significantly decreased the folded stability of OVCA2 (H206A Δ*T*_M_ = - 10°C) and introduced multiple stable folded intermediates (Fig 3B). All three variants were, however, stable at the temperature used for kinetic measurements (23°C).

The relative catalytic activity of each variant was measured to confirm their role in catalysis. Substitution of each proposed catalytic residue individually with alanine inhibited the catalytic activity of OVCA2 to long chain alkyl ester substrates (Figs 3C and 3D and S3). Substitution of the serine nucleophile (Ser117) and histidine base (His206) completely inactivated OVCA2 to background hydrolysis rates (S3 Fig and S4 Table), whereas the D179A variant maintained a low level of enzymatic activity against all four *p*-nitrophenyl substrates (S3 Fig and S4 Table). The decreased importance of the acidic amino acid in the catalytic triad have been observed for other serine hydrolases.[40, 50, 51] Together, OVCA2 was found to utilize a classic catalytic triad of Ser117-His206-Asp179 to perform its hydrolysis reactions, further confirming its assignment as a metabolic serine hydrolase.

### Comparative substrate specificity of FSH1 and OVCA2

Without pure, active OVCA2 protein, previous information about OVCA2 had been inferred based on analogy to the *S*. *cerevisiae* homolog FSH1.[17, 22] As the 21% similarity between human OVCA2 and FSH1 (Fig 1A) falls within the percent similarity range where direct functional and structural homology is difficult to judge, we wanted to confirm this homology assignment using direct biochemical metrics.[52, 53] To perform this comparison, we purified and expressed FSH1 in *E*. *coli* (S1 Fig). Purified FSH1 was well-folded and had a higher thermal stability than OVCA2 (Δ*T*_M_ = + 10°C; S1 and S2 Figs). FSH1 expression and purification was also performed in the more traditional *E*. *coli* BL21 (DE3) pLyS strain with standard bacterial growth media (LB), suggesting greater intrinsic stability to FSH1 (S1 Fig).

**Fig 4:**
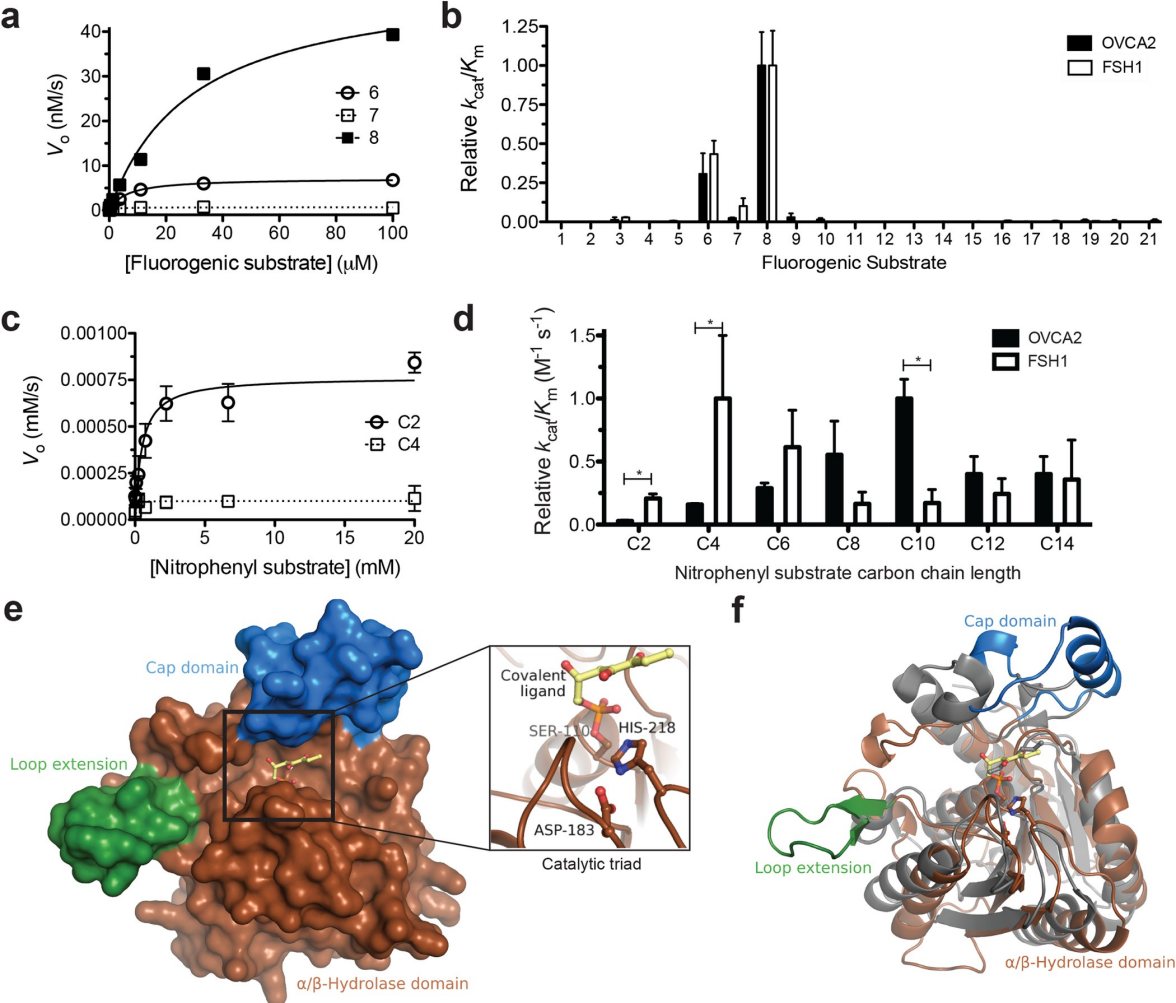
Comparative activity of FSH1. (A) Kinetic activity of FSH1 against substrates **6** (open circles), **7** (open squares), and **8** (closed squares). All measurements completed in triplicate and shown ± SD. (B) Global comparison of the relative catalytic specificity (*k*_cat_/*K*_M_) of FSH1 and OVCA2 against each of the 21 fluorogenic substrates (structures and numbering given in Fig 2A). Catalytic specificities were normalized based on the highest activity substrate for each enzyme. All relative *k*_cat_/*K*_M_ values for OVCA2 and FSH1 are not significantly different at *p* < 0.05 (Unpaired two-tailed t-test; GraphPad Prism 5). Detailed kinetic values are given in S2 and S3 Tables. (C) Kinetic activity of FSH1 against *p-*nitrophenyl acetate (C2) and *p-*nitrophenyl butyrate (C4). Data points were fitted to the Michaelis-Menten equation and are shown ± SD. (D) Relative catalytic efficiency comparison of FSH1 and OVCA2 against *p*-nitrophenyl substrates. Catalytic specificities were normalized based on the highest activity substrate for each enzyme. Relative catalytic efficiency values (*k*_cat_/*K*_m_) are given ± SD. Values designated with an asterisk (*) were significantly different than each other at *p* < 0.05 (Unpaired two-tailed t-test; GraphPad Prism 5). Detailed kinetic data are provided in S2 and S3 Tables. (E) Binding pocket structure of FSH1 (PDB: 1YCD). The α/β hydrolase domain is shown in brown with extensions from the basic domain, including the cap domain (blue) and loop extension (green), colored separately. Inset: The proposed catalytic triad of FSH1 with unknown bound ligand, hypothetically labeled as 2-hydroxy-4,5-dioxoheptyl hydrogen phosphonate.[22] (F) Comparative alignment of FSH1 (brown) to its closest structural homolog in the PDB (grey), a carboxyl esterase from *R*. *sphaeroides* (p-value = 4.2x10^-9^; RMSD = 2.84 Å). Structures aligned using the RCSB pre-calculated alignments.[63].

The substrate specificity of purified FSH1 was then comprehensively characterized against the same two ester substrate libraries as OVCA2 (Fig 2A). Like OVCA2, FSH1 showed Michaelis-Menten kinetics with both substrate libraries (Fig 4A and 4C). Overlapping with OVCA2, FSH1 showed highest activity against the alkyl ether ester fluorogenic substrates (**6–8**) and statistically equivalent (*p* < 0.05) relative ratios as OVCA2 (Fig 4B). The absolute values for the catalytic efficiency (*k*_cat_/*K*_M_) of FSH1 for the fluorogenic substrates were however >10-fold higher than OVCA2 with highest activity for substrate **8** (4300 ± 1000 M^-1^ s^-1^; S3 Table), suggesting greater structural plasticity for FSH1.[36] The higher activity of FSH1 toward shorter ester substrates was then reaffirmed with the chromogenic *p*-nitrophenyl ester substrates (Fig 4D), as FSH1 showed highest activity toward four- and six-carbon *p*-nitrophenyl alkyl ester substrates. FSH1 maintained fairly broad substrate promiscuity toward the *p*-nitrophenyl ester substrates with greater than >2000 M^-1^ s^-1^ catalytic efficiency toward substrates from 2-carbons to 14-carbons (S3 Table). Overall, FSH1 showed overlapping substrate specificity with OVCA2 for fluorogenic substrates but slightly diverged from OVCA2 for the *p*-nitrophenyl ester substrates with increased activity for shorter substrates (C2 and C4) and less relative activity for longer substrates (C10).

The broad substrate specificity of FSH1 is likely correlated with its shallow, broad binding pocket (Fig 4E).[23] The structure of FSH1 showcased a compact α/β hydrolase protein fold with a small cap domain overlapping the binding pocket and a short β-sheet extension off one end of the binding pocket (Fig 4E).[22] The binding pocket was mostly hydrophobic and fairly solvent exposed with a narrow groove wrapped around the cap domain. Structural alignment of FSH1 to its closest structural homolog (PDB: 4FHZ) shows that its cap domain architecture and loop extension are unique to FSH1 (Fig 4F). Cap domains are a major determinant in differentiating serine hydrolase substrate specificity and the variation in cap domains between FSH1 and structural homologues suggests unique substrates for FSH1 and by analogy OVCA2.[43, 54] However, like FSH1, the aligned structural homolog from *Rhodobacter spahaeroides* (PDB: 4FHZ) accepted a wide range of *p*-nitrophenyl and other structural esters, indicating that the overall α/β hydrolase architecture shared by FSH1 is fairly promiscuous.[55]

In the structural model of OVCA2,[17] the broad binding pocket of FSH1 was narrowed to a longer, enclosed substrate binding pocket (Fig 4F). This more enclosed pocket of OVCA2 likely selects against shorter alkyl esters and this greater selectivity filter is reflected in the substrate selectivity of OVCA2 (Fig 4B and 4D). The binding pocket and cap structure of FSH1 do however resemble the structures of the acyl protein thioesterase (APT) superfamily, which are the closest human structural homologues of FSH1 (S4 Fig).[17, 23] APTs are also metabolic serine hydrolases with long, narrow hydrophobic binding pockets that do not undergo structural rearrangement upon substrate binding and perform diverse biological roles.[6, 56, 57] Reinforcing this comparison of APTs to FSH1 and OVCA2, the most well-characterized human APT (APT1) had low activity toward a library of small fluorogenic esters, but robust activity toward extended chain *p*-nitrophenyl esters.[6, 58] A similar narrowing of substrate specificity was also observed in bacterial versus human APTs where human APT like OVCA2 showed higher selectivity against for longer chain ester substrates.[6, 58]

Overall, based on substrate comparison, FSH1 provides a good preliminary model for OVCA2, but its substrate specificity and structure reflect broadened substrate binding and reactivity than OVCA2. Further refinement of these substrate specificity differences between FSH1 and OVCA2 will require a three-dimensional structure of OVCA2. Overlapping structural and enzymatic characteristics, however, indicate that OVCA2 and FSH1 share substrate binding characteristics with the APT superfamily of human metabolic serine hydrolases.

## Conclusions

OVCA2 is a human metabolic serine hydrolase with links to cancer proliferation and acetaldehyde remediation. OVCA2 is a well-folded protein (*T*_M_ = 47 ± 0.9°C) but required systematic optimization for heterologous bacterial expression (Fig 1). Across two ester libraries, OVCA2 strongly selected for unbranched, alkyl ester substrates, preferring substrates greater than 10-carbons (Fig 2), using a classic catalytic triad of Ser117-His206-Asp179 to perform these hydrolysis reactions (Fig 3). Comparison of OVCA2 to the proposed structural and functional homologue FSH1 from *S*. *cerevisiae* illustrated the increased folded stability (Δ*T*_M_ = + 10°C) and substrate promiscuity (Fig 4) of FSH1, especially toward smaller fluorogenic substrates. Yet, confirmed their overall homology based on similar catalytic preference for straight-chain extended alkyl substrates (Fig 4). FSH1 is a reasonable preliminary structural and enzymatic model for OVCA2, but direct investigation of OVCA2 is necessary to define its three-dimensional structure and biological substrates.

## Materials and methods

### Purification of OVCA2 from *H*. *sapiens*

A Gateway donor plasmid (pDONR223) containing the *OVCA2* gene from *Homo sapiens* (Genbank: NP_543012.1; protein name OVCA2) was obtained from the DNASU Plasmid Repository (Clone ID: HsCD00352893). The *OVCA2* gene was then recombined into the bacterial expression plasmid pDEST17 by Gateway cloning (ThermoFisher Scientific) using LR clonase. A terminal stop codon was added by Quikchange site-directed mutagenesis (Agilent) using the manufacturer’s suggested protocol and the primer given in S6 Table (Integrated DNA Technologies). The final bacterial expression plasmid (pDEST17-OVCA2) was transformed into *E*. *coli* Origami B (DE3) pLyS cells (EMD Millipore) and protein was expressed using the EnPresso B expression system (Sigma-Aldrich). For this procedure, a small LB culture (2 mL) containing ampicillin (200 μg/mL), kanamycin (20 μg/mL), chloramphenicol (15 μg/mL), and tetracycline (7.5 μg/mL) was inoculated from a freshly transformed plate of *E*. *coli* Origami B (DE3) pLyS (pDEST17-OVCA2) and allowed to grow for 8 hours at 37°C and 225 rpm. The small culture was then used to seed a 50 mL culture containing all four antibiotics in EnPresso media and the larger culture was grown for 16–18 hours at 30°C and 250 rpm. IPTG (100 μM), booster tablets, and reagent A were then added and induction proceeded for 24 hours at 30°C and 250 rpm. Bacterial cultures were collected by centrifugation at 6,000 × g for 10 min at 4°C. For wild-type OVCA2, three 50 mL EnPresso expression cultures were completed in parallel and were combined into single bacterial cell pellets that were resuspended in PBS (30 mL) containing β-mercaptoethanol (BME; 5 mM) and stored at -20°C.

To disrupt the bacterial cell wall, lysozyme (250 mg) and Bug Buster solution (2.0 mL of 10X; EMD Millipore) were added and cell lysis proceeded on a rotating shaker for 2 h at 4°C. To remove insoluble cell material, lysed cells were centrifuged at 16,000 x g for 10 min at 4°C. Ni-NTA agarose (1.0 mL; Qiagen, Valencia, CA) was added to the soluble fraction and allowed to incubate at 4°C for 15 min. The resin was washed three times with PBS + BME (5 mM) containing increasing concentrations of ice-cold imidazole (40 mL each of PBS containing 10 mM imidazole, 25 mM imidazole, or 50 mM imidazole) and recollected by centrifugation at 1000 × g for 1 min at 4°C between wash steps. OVCA2 was eluted in PBS containing imidazole (500 mM) and dialyzed against PBS containing dithiothreitol (DTT; 5 mM) overnight at 4°C with constant stirring (10K MWCO; ThermoFisher Scientific).

The purity of OVCA2 was confirmed by SDS–PAGE on a 4–20% gradient gel (Fig 1). The expected molecular weight of the OVCA2 protein was 27.0 kD. The concentration of OVCA2 was determined by measuring the absorbance at 280 nm and by calculating the extinction coefficient (ε^280^ = 16960 M^–1^ s^–1^ with all cysteines reduced) on Expasy.

### Site-directed mutagenesis of OVCA2 and purification of OVCA2 variants

Variants of OVCA2 were produced by QuikChange II site-directed mutagenesis of the pDEST17-OVCA2 template plasmid DNA using the manufacturer’s suggested procedure (Agilent), the only exception being specific annealing temperatures and the mutagenesis primers (Integrated DNA Technologies) outlined in S6 Table. Proper mutations in the *OVCA2* DNA sequence were confirmed by DNA sequencing (Genewiz) using T7 and/or T7-terminal sequencing primers. Plasmids coding for *OVCA2* variants were transformed into *E*. *coli* Origami B pLyS (DE3) cells and variants of OVCA2 were overexpressed, purified, quantitated, and characterized using the same procedure as for wild-type OVCA2, except only one equivalent of EnPresso expression system was used per OVCA2 variant.

### Purification of FSH1 from *S*. *cerevisiae*

A Gateway donor plasmid (pDONR201) containing the *FSH1* gene from *Saccharomyces cerevisiae* (NP_014923.1; protein name FSH1) was obtained from the DNASU Plasmid Repository (Clone ID: ScCD00009796). The *FSH1* gene containing an in-frame stop codon was then recombined into the bacterial expression plasmid pDEST17 by Gateway cloning (ThermoFisher Scientific) using LR clonase. This bacterial plasmid (pDEST17-FSH1) was transformed into *E*. *coli* BL21 (DE3) pLyS cells (Agilent). A saturated overnight culture of *E*. *coli* BL21 (DE3) pLyS (pDEST17-FSH1) in LB media containing ampicillin (200 μg/mL) and chloramphenicol (30 μg/mL) was used to inoculate LB-media (1L) containing ampicillin (100 μg/mL) and chloramphenicol (30 μg/mL) and the bacterial culture was grown with constant shaking (225 rpm) at 37°C. When the OD_600_ reached 0.6–0.8, the temperature of the culture was decreased to 23°C and isopropyl β-D-1-thiogalactopyranoside (IPTG) was added to a final concentration of 0.5 mM. Protein induction proceeded for 16–20 hours at 23°C. Bacterial cultures were collected by centrifugation at 6,000 × g for 10 min at 4°C. The bacterial cell pellet was resuspended in PBS (40 mL) and stored at -20°C.

To disrupt the bacterial cell wall, lysozyme (250 mg) and Bug Buster solution (4.0 mL of 10X; EMD Millipore) were added and cell lysis proceeded on a rotating shaker for 2 h at 4°C. To remove insoluble cell material, lysed cells were centrifuged at 16,000 x g for 10 min at 4°C. Ni-NTA agarose (1.0 mL; Qiagen, Valencia, CA) was added to the soluble fraction and allowed to incubate at 4°C for 15 min. The resin was washed three times with PBS containing increasing concentrations of ice-cold imidazole (40 mL each of PBS containing 10 mM imidazole, 25 mM imidazole, or 50 mM imidazole) and recollected by centrifugation at 1000 × g for 1 min at 4°C between wash steps. FSH1 was eluted in PBS containing imidazole (250 mM) and dialyzed against PBS overnight at 4°C with constant stirring (10K MWCO; Pierce, Rockford, IL).

The purity of FSH1 was confirmed by SDS–PAGE on a 4–20% gradient gel (S1 Fig). The expected molecular weight of the FSH1 protein was 27.3 kD. The concentration of FSH1 was determined by measuring the absorbance at 280 nm and by calculating the extinction coefficient (ε^280^ = 24410 M^–1^ s^–1^ with all free cysteines) on Expasy.

### Ester hydrolase substrates

*p*-nitrophenyl substrates were from Sigma-Aldrich. Compounds **1**–**21** (Fig 2) were synthesized as described previously.[34–36, 40]

### Kinetic measurements with fluorogenic ester substrates

The enzymatic activity of OVCA2, OVCA2 variants, and FSH1 was measured against the fluorogenic ester substrates (Fig 2) using a 96-well microplate assay.[36, 37] Fluorogenic substrates were prepared as stock solutions in DMSO (10 mM) and were diluted into PBS containing acetylated BSA (PBS–BSA; 0.1 mg/mL) to starting concentrations between 10–100 μM, depending on the *K*_m_ value of each enzyme for the substrate. The majority of the substrates (substrates **1–21** for OVCA2 and substrates **6–8**; **19–21** for FSH1) had the same starting concentration (100 μM) with substrates **1–5** and **9–18** for FSH1 (10 μM) requiring lower starting concentrations. Eight serial 3-fold dilutions (60 μL into 180 μL total volume) of each substrate were made using PBS–BSA. Fluorogenic substrate dilutions (95 μL) were then transferred to a black 96-well microplate (Corning, Lowell, MA).

Protein (5 μL; final concentration OVCA2 = 263 nM; FSH1 = 275 nM) was added to the diluted fluorogenic substrates in the 96-well microplate (100 μL final volume) and the fluorescence change (λ_ex_ = 485 nm, λ_em_ = 528 nm) was measured for 7.5 min and intervals of 50 sec at 25°C on a Biotek Synergy H1 Multimode plate reader (Biotek Instruments). The fluorescence change was converted to molar concentrations using a fluorescein standard curve (30 nM–0.23 nM for 10 μM starting substrate concentrations and 300 nM–2.3 nM for 100 μM starting concentrations), whose fluorescence was measured simultaneously. The initial rates of the reactions were measured in triplicate and plotted versus fluorogenic enzyme substrate concentration. The saturation enzyme kinetic traces were fitted to a standard Michaelis–Menten equation using Origin 6.1 (OriginLab Corp., Northhampton, MA) and values for *k*_cat_, *K*_M_ and *k*_cat_/*K*_M_ calculated.

### Kinetic measurements with p-nitrophenyl substrates

The enzymatic activity of OVCA2, OVCA2 variants, and FSH1 was measured against *p*-nitrophenyl acetate (C2), *p*-nitrophenyl butyrate (C4), *p*-nitrophenyl valerate (C6), *p*-nitrophenyl octanoate (C8), *p*-nitrophenyl decanoate (C10), *p*-nitrophenyl laurate (C12), and *p*-nitrophenyl myristate (C14) (Sigma–Aldrich) using a 96-well microplate assay (Fig 2).[36, 42] All seven substrates, *p*-nitrophenyl acetate and *p*-nitrophenyl butyrate (2 M), *p*-nitrophenyl valerate, *p*-nitrophenyl octanoate, *p*-nitrophenyl decanoate, *p*-nitrophenyl laurate, and *p*-nitrophenyl myristate (200 mM) were prepared as stock solutions in acetonitrile and diluted into PBS containing acetylated BSA (PBS–BSA; 0.1 mg/mL). Similar to fluorogenic substrates (Fig 2), the starting substrate concentrations had to be adjusted based on variations in *K*_M_ values and substrate solubility. For OVCA2 and FSH1, the starting concentration for *p*-nitrophenyl acetate and *p*-nitrophenyl butyrate was 20 mM, for *p*-nitrophenyl valerate and *p*-nitrophenyl octanoate was 2 mM, and for *p*-nitrophenyl decanoate, *p*-nitrophenyl laurate, and *p*-nitrophenyl myristate was 0.5 mM. Eight serial 2-fold dilutions (110 μL into 220 μL total volume) were made using PBS–BSA containing 1% acetonitrile. Substrate dilutions (95 μL) were transferred to a clear 96-well microplate and enzyme (5 μL; final concentration OVCA2 = 263 nM; FSH1 = 550 nM) was added to start the reaction. The absorbance change at 412 nm was measured on a Biotek Synergy H1 Multimode plate reader for 4 min and 15 sec intervals at 25°C. The change in absorbance was converted to molar concentrations using the extinction coefficient of *p*-nitrophenol (Δε_412_ = 1.034 mM^–1^ cm^–1^).[59] The initial rates of the reactions were measured in triplicate and plotted versus *p*-nitrophenyl substrate concentration. The saturation enzyme kinetic traces were fitted to a standard Michaelis–Menten equation using Origin 6.1 (OriginLab Corp., Northhampton, MA) and values for *k*_cat_, *K*_M_ and *k*_cat_/*K*_M_ calculated.

### Thermal stability measurement

Similar to previously published methods, the thermal stability of OVCA2, variants of OVCA2, and FSH1 was determined using differential scanning fluorimetry (DSF).[36, 60] Proteins (0.3 mg/mL) were diluted in at least triplicate in PBS containing a 1:250 dilution of SYPRO Orange (ThermoFischer Scientific). The samples were heated from 15°C to 85°C at 1.0°C/min in a thermocycler (Bio-rad C1000 Thermocycler with CFX96 Real-time System, Hercules, CA) and the change in SYPRO Orange fluorescence followed over time ((λ_ex_ = 450–490 nm, λ_em_ = 610–650 nm). The melting temperature (*T*_m_) was determined by plotting the first derivative of fluorescence versus temperature and finding the temperature at the midpoint of the transition. As in previous analyses,[36, 42, 61] all graphs were normalized so that minimum fluorescence was set to 0 and maximum fluorescence set to 1.

### Detection of thiol groups

Nbs_2_ reacts with free thiol groups but not disulfide bonds to produce a yellow chromophore whose absorbance at 412 nm can be used to calculate the number of free thiol groups.[31, 32] Purified OVCA2 (0.25 mg/mL; 0.00926 mM) was dialyzed back into PBS without DTT and incubated in triplicate on a clear microplate (50 μL total volume) with a 10-fold molar excess of Nbs_2_ in PBS for 30 min at 25°C. The number of free thiols was determined by UV absorption at 412 nm on a Biotek Synergy H1 Multimode plate reader using *ε*_412 nm_ = 14.15 × 10^3^ M^-1^ s^-1^.[32] Background Nbs_2_ absorbance was subtracted based on identical measurements with only PBS buffer.

### Phylogenetic analysis of OVCA2

The amino acid sequence of OVCA2 was aligned using Clustal Omega (EMBL EBI). A cladogram of the aligned proteins was then constructed using Drawgram from the Mobyle Pasteur (Fig 1 and S1 Table). The catalytic triad amino acids were extracted from the alignment based on sequence conservation and the presence of the catalytic motif (G-x-S-x-G). The sequences used in the alignment were from *Homo sapiens* (NP_543012.1), *Macaca fascicularis* (XP_005582523.1), *Mus musculus* (NP_081412.1), *Xenopus laevis* (XP_018104262.1), *Danio rerio* (NP_001018391.1), *Caenorhabditis elegans* (NP_502376.1), *Drosophila melanogaster* (NP_650895.1), *Schizosaccharomyces pombe* (NP_588353.1), *Saccharomyces cerevisiae* FSH1 (NP_011915.1), *Saccharomyces cerevisiae* FSH2 (NP_013949.1), *Saccharomyces cerevisiae* FSH3 (NP_014923.1). Sequences for alignment were chosen based on protein BLAST analysis of OVCA2 and extracting unique protein sequences from model organisms with significant percent similarity (> 20%).

## Supporting information

S1 Table Multiple sequence alignment of OVCA2. (DOCX)

S2 Table Kinetic characterization of OVCA2. (DOCX)

S3 Table Kinetic characterization of FSH1. (DOCX)

S4 Table Biochemical characterization of OVCA2 active site variants. (DOCX)

S5 Table Multiple sequence alignment of OVCA2, FSH1, and human APTs. (DOCX)

S6 Table PCR primers used for site-directed mutagenesis. (DOCX)

S1 Fig Purification of FSH1. An SDS–PAGE gel (4–20%) showing the protein purification of FSH1. Representative purification samples (10, 25, and 50 mM imidazole washes) shown. FSH1 was purified according to the procedure outlined in Experimental Procedures. The expected molecular weight of FSH1 is 27.3 kDa. The molecular weight was confirmed by comparison to the Kaleidoscope prestained protein standard (Bio-rad laboratories).
(DOCX)

S2 Fig Thermal stability and folding of FSH1. The folded to unfolded transition for FSH1 (0.3 mg/mL in PBS) was observed by DSF. The measurement was completed in triplicate and is shown ± SD. The majority of the error is smaller than the size of the data marker.
(DOCX)

S3 Fig Hydrolysis reactions of OVCA2 variants versus background hydrolysis rates. Kinetic activity of active site OVCA2 variants (S117A closed circles, D179A open diamonds, and H206A closed squares) compared to the background hydrolysis rate of the same four chromogenic substrates in PBS measured identically. Comparative kinetic activity against a) *p-*nitrophenyl octanoate (C8), b) *p-*nitrophenyl decanoate (C10), c) *p-*nitrophenyl laurate (C12), and d) *p-*nitrophenyl myristate (C14). Data points were fitted to the Michaelis-Menten equation and are shown ± SD. Each of these plots show that the active site variants for S117A and H206A have activity below the background hydrolysis rate, confirming that substitution of these residues with alanine completely inactivates OVCA2. The D179A variant however shows residual catalytic activity above background hydrolysis rates.
(DOCX)

S4 Fig Structural alignment of FSH1 and human APTs. (A and B) Comparative alignment of FSH1 (brown; PDB ID: 1YCD) to its closest human structural homolog in the PDB (teal; PDB ID: 3U0V), human LYPLAL1 (p-value = 9.8x10^-9^; RMSD = 3.83 Å). Cartoon representation in A with the surface of FSH1 shown in B. (C and D) Comparative alignment of FSH1 (brown; PDB ID: 1YCD) to the second closest human structural homolog in the PDB (grey; PDB ID: 1FJ2), human APT1 (p-value = 2.5x10^-8^; RMSD = 3.13 Å). Cartoon representation in C with the surface of FSH1 shown in D. (E) Close up view of the FSH1 and LYPLAL1 active site surface. The covalent ligand bound to the active site of FSH1 is shown in yellow sticks. The overlap of the LYPLAL1 teal surface with the FSH1 bound ligand shows that the binding surface of LYPLAL1 is shallower than FSH1. (F) Close up view of the APT1 surface (grey; PDB ID: 5SYM) with FSH1 shown in cartoon. The long, open hydrophobic binding pocket of APT1 is shown by the bound ML348 inhibitor, which differs from the closed off pocket of FSH1 (B). All structures aligned using the RCSB pre-calculated alignments.[63]
(DOCX)
