# Supplementary material for: Comparative analysis of the human serine hydrolase OVCA2 to the model serine hydrolase homolog FSH1 from S. cerevisiae
Source: PLoS One. 2020 Mar 17;15(3):e0230166. doi: 10.1371/journal.pone.0230166 (PMC7077851; doi:10.1371/journal.pone.0230166)
Supplement: S2 Table — (DOCX) [file pone.0230166.s002.docx]

| S2 Table: Kinetic characterization of OVCA2. | | | |
| --- | --- | --- | --- |
| Substrate | *k*_cat_ (s^-1^) | *K*_m_ (mM) | *k*_cat_/*K*_m_ (M^-1^ s^-1^) |
| **C2^a^** | 0.26 ± 0.03 | 1.4 ± 0.6 | 200 ± 50 |
| **C4** | 0.30 ± 0.03 | 2.9 ± 0.1 | 1100 ± 50 |
| **C6** | 0.30 ± 0.02 | 0.16 ± 0.04 | 2000 ± 550 |
| **C8** | 0.023 ± 0.005 | 0.0061 ± 0.0057 | 3800 ± 1800 |
| **C10** | 0.025 ± 0.001 | 0.00384 ± 0.0011 | 6800 ± 1000 |
| **C12** | 0.076 ± 0.01 | 0.029 ± 0.019 | 2700 ± 950 |
| **C14** | 0.076 ± 0.01 | 0.017 ± 0.014 | 4500 ± 1800 |
|  | *k*_cat_ (10^-3^ s^-1^) | *K*_m_ (µM) | *k*_cat_/*K*_m_ (M^-1^s^-1^) |
| **1^b^** | 0.011 ± 0.001 | 21 ± 4 | 0.52 ± 0.11 |
| **2** | 0.0018 ± 0.0003 | 3.1 ± 2.2 | 0.47 ± 0.07 |
| **3** | 0.0018 ± 0.0005 | 0.43 ± 0.57 | 4.1 ± 5.7 |
| **4** | 0.0060 ± 0.0016^d^ | > 25^c^ | ND |
| **5** | 0.0011 ± 0.0001 | 0.47 ± 0.09 | 2.2 ± 0.4 |
| **6** | 1.3 ± 0.2 | 12 ± 5 | 100 ± 40 |
| **7** | 0.0047 ± 0.0002 | 0.57 ± 0.08 | 8.2 ± 1.2 |
| **8** | 1.2 ± 0.1 | 3.7 ± 0.8 | 330 ± 70 |
| **9** | 0.00043 ± 0.00004^d^ | < 0.1^c^ | ND |
| **10** | 0.0025 ± 0.0004 | 0.51 ± 0.35 | 4.8 ± 3.4 |
| **11** | 0.012 ± 0.004^d^ | > 25^c^ | ND |
| **12** | 0.0027 ± 0.0002 | 8.2 ± 1.9 | 0.33 ± 0.08 |
| **13** | 0.0018 ± 0.0002 | 2.5 ± 0.8 | 0.73 ± 0.26 |
| **14** | 0.021 ± 0.006^d^ | > 25^c^ | ND |
| **15** | 0.00012 ± 0.00001 | 5.6 ± 1.4 | 0.21 ± 0.05 |
| **16** | 0.042 ± 0.005^d^ | > 25^c^ | ND |
| **17** | 0.00090 ± 0.00008 | 0.94 ± 0.36 | 0.95 ± 0.37 |
| **18** | 0.0013 ± 0.0001 | 0.61 ± 0.23 | 1.9 ± 0.8 |
| **19** | 0.045 ± 0.008 | 13 ± 7 | 3.4 ± 2.0 |
| **20** | 0.0015 ± 0.0024 | 10 ± 6 | 1.4 ± 2.5 |
| **21** | 0.0079 ± 0.0007 | 24 ± 6 | 0.33 ± 0.09 |
| ^a^Kinetic constants for *p*-nitrophenyl substrates were determined by measuring the change in A_412_ due to ester hydrolysis. Substrates represent different carbon chain lengths: *p*-nitrophenyl acetate (C2), *p*-nitrophenyl butyrate (C4), *p*-nitrophenyl valerate (C6), *p*-nitrophenyl octanoate (C8), *p*-nitrophenyl decanoate (C10), *p*-nitrophenyl laurate (C12), and *p*-nitrophenyl myristate (C14).  ^b^Kinetic constants for substrates **1–21** were determined by measuring the increase in fluorogenic enzyme substrate fluorescence over time. Data were fitted to a standard Michaelis-Menten equation to determine the values for *k*_cat_, *K*_M_, and *k*_cat_/*K*_M_. Kinetic measurements for each substrate were repeated three times and the values are given ± SD.  ^c^Due to the low activity of OVCA2 against these fluorogenic substrates, *K*_M_ values were not able to determined accurately. Upper (> 25 µM) and lower (> 0.1 µM) limits for *K*_M_ values were set based on substrate dilution ranges.  ^d^Values for *k*_cat_ are derived from fitting to the Michaelis-Menten equation. Due to the uncertainty in *K*_M_, these *k*_cat_ values are also approximations. | | | |
