# Supplementary material for: Comparative analysis of the human serine hydrolase OVCA2 to the model serine hydrolase homolog FSH1 from S. cerevisiae
Source: PLoS One. 2020 Mar 17;15(3):e0230166. doi: 10.1371/journal.pone.0230166 (PMC7077851; doi:10.1371/journal.pone.0230166)
Supplement: S3 Table — (DOCX) [file pone.0230166.s003.docx]

| S3 Table: Kinetic characterization of FSH1. | | | | |
| --- | --- | --- | --- | --- |
| Substrate | *k*_cat_ (s^-1^) | *K*_m_ (mM) | *k*_cat_/*K*_m_ (M^-1^ s^-1^) |  |
| **C2^a^** | 1.4 ± 0.1 | 0.49 ± 0.17 | 2900 ± 500 |  |
| **C4** | 0.16 ± 0.02 | 0.011 ± 0.011 | 14000 ± 7000 |  |
| **C6** | 0.15 ± 0.03 | 0.017 ± 0.016 | 8600 ± 4100 |  |
| **C8** | 0.10 ± 0.02 | 0.043 ± 0.046 | 2300 ± 1300 |  |
| **C10** | 0.046 ± 0.013 | 0.019 ± 0.023 | 2400 ± 1500 |  |
| **C12** | 0.11 ± 0.03 | 0.032 ± 0.031 | 3400 ± 1700 |  |
| **C14** | 0.061 ± 0.024 | 0.012 ± 0.02 | 5000 ± 4400 |  |
|  | *k*_cat_ (10^-3^ s^-1^) | *K*_m_ (µM) | *k*_cat_/*K*_m_ (M^-1^s^-1^) |  |
| **1^b^** | 0.0076 ± 0.0001 | 1.33 ± 0.1 | 5.7 ± 0.4 |  |
| **2** | 0.016 ± 0.002 | 5.3 ± 1.6 | 3.0 ± 1.0 |  |
| **3** | 0.042 ± 0.001 | 0.35 ± 0.03 | 120 ± 10 |  |
| **4** | 0.033 ± 0.004 | 3.5 ± 1.0 | 9.7 ± 3.0 |  |
| **5** | 0.033 ± 0.001 | 3.2 ± 0.3 | 10 ± 1 |  |
| **6** | 30 ± 2 | 16 ± 3 | 1900 ± 400 |  |
| **7** | 2.8 ± 0.4 | 6.4 ± 3.0 | 440 ± 210 |  |
| **8** | 230 ± 50^d^ | > 25^c^ | ND |  |
| **9** | 0.00033 ± 0.00007 | 0.27 ± 0.26 | 1.2 ± 1.2 |  |
| **10** | 0.025 ± 0.002 | 4.7 ± 0.7 | 5.2 ± 0.8 |  |
| **11** | 0.042 ± 0.003 | 7.9 ± 1.1 | 5.3 ± 0.8 |  |
| **12** | 0.22 ± 0.01^d^ | > 25^c^ | ND |  |
| **13** | 0.17 ± 0.25^d^ | > 25^c^ | ND |  |
| **14** | 0.00077 ± 0.00011 | 2.1 ± 0.8 | 0.36 ± 0.14 |  |
| **15** | 0.00017 ± 0.00002^d^ | < 0.1^c^ | ND |  |
| **16** | 0.31 ± 0.1 | 9.3 ± 1.6 | 33 ± 6 |  |
| **17** | 0.013 ± 0.005^d^ | > 25^c^ | ND |  |
| **18** | 0.0013 ± 0.0003 | 1.15 ± 0.87 | 1.1 ± 0.9 |  |
| **19** | 1.7 ± 0.1^d^ | > 25^c^ | ND |  |
| **20** | 0.13 ± 0.01^d^ | > 25^c^ | ND |  |
| **21** | 0.17 ± 0.06^d^ | > 25^c^ | ND |  |
| ^a^Kinetic constants for *p*-nitrophenyl substrates were determined by measuring the change in A_412_ due to ester hydrolysis. Substrates represent different carbon chain lengths: *p*-nitrophenyl acetate (C2), *p*-nitrophenyl butyrate (C4), *p*-nitrophenyl valerate (C6), *p*-nitrophenyl octanoate (C8), *p*-nitrophenyl decanoate (C10), *p*-nitrophenyl laurate (C12), and *p*-nitrophenyl myristate (C14).  ^b^Kinetic constants for substrates **1–21** were determined by measuring the increase in fluorogenic enzyme substrate fluorescence over time. Data were fitted to a standard Michaelis-Menten equation to determine the values for *k*_cat_, *K*_M_, and *k*_cat_/*K*_M_. Kinetic measurements for each substrate were repeated three times and the values are given ± SD.  ^c^Due to the low activity of FSH1 against these fluorogenic substrates, *K*_M_ values were not able to determined accurately. Upper (> 25 µM) and lower (> 0.1 µM) limits for *K*_M_ values were set based on substrate dilution ranges.  ^d^Values for *k*_cat_ are derived from fitting to the Michaelis-Menten equation. Due to the uncertainty in *K*_M_, these *k*_cat_ values are also approximations. | | | | |
