# Supplementary material for: Comparative analysis of the human serine hydrolase OVCA2 to the model serine hydrolase homolog FSH1 from S. cerevisiae
Source: PLoS One. 2020 Mar 17;15(3):e0230166. doi: 10.1371/journal.pone.0230166 (PMC7077851; doi:10.1371/journal.pone.0230166)
Supplement: S4 Table — (DOCX) [file pone.0230166.s004.docx]

|  | S4 Table: Biochemical characterization of OVCA2 active site variants. | | | | |
| --- | --- | --- | --- | --- | --- |
| OVCA2 variant | | *k*_cat_  (10^-3^ s^-1^) ^a^ | *K*_m_  (µM) | *k*_cat_/*K*_m_  (M^–1^s^–1^) | *T*_m_  (°C)^b^ |
| *S117A* | |  |  |  | 50.0 ± 0.9 |
| C8 | | ND ^c^ | ND | ND |  |
| C10 | | ND | ND | ND |  |
| C12 | | ND | ND | ND |  |
| C14 | | ND | ND | ND |  |
| *H206A* | |  |  |  | 37.0 ± 0.9 |
| C8 | | ND | ND | ND |  |
| C10 | | ND | ND | ND |  |
| C12 | | ND | ND | ND |  |
| C14 | | ND | ND | ND |  |
| *D179A* | |  |  |  | 49.0 ± 0.9 |
| C8 | | 0.0010 ± 0.0006 | 2.0 ± 0.2 | 0.50 ± 0.30 |  |
| C10 | | 0.037 ± 0.004 | 3.2 ± 1.5 | 11 ± 3 |  |
| C12 | | 0.073 ± 0.008 | 1.1 ± 0.6 | 69 ± 20 |  |
| C14 | | 0.046 ± 0.004 | 5.7 ± 1.9 | 7.8 ± 1.4 |  |
| ^a^Kinetic constants for *p*-nitrophenyl substrates were determined by measuring the change in A_412_ due to ester hydrolysis. Substrates represent the most active substrates for wild-type OVCA2: *p*-nitrophenyl octanoate (C8), *p*-nitrophenyl decanoate (C10), *p*-nitrophenyl laurate (C12), and *p*-nitrophenyl myristate (C14).  ^b^ Values for *T*_m_ were determined by following the change in Sypro Orange fluorescence with increasing temperature. Melting curves were repeated three times for each variant and the *T*_m_ values reported ± SD.  ^c^ Kinetic constants for these active site variants were not determinable (ND) as the values were within error of the background hydrolysis of each substrate in the same buffer under the same conditions. Kinetic curves comparing the catalytic activity of these variants against each substrate versus the hydrolysis rates are shown in Figure S3. | | | | | |
