# Supplementary material for: Comparative analysis of the human serine hydrolase OVCA2 to the model serine hydrolase homolog FSH1 from S. cerevisiae
Source: PLoS One. 2020 Mar 17;15(3):e0230166. doi: 10.1371/journal.pone.0230166 (PMC7077851; doi:10.1371/journal.pone.0230166)
Supplement: S6 Table — (DOCX) [file pone.0230166.s006.docx]

| S6 Table: PCR primers used for site-directed mutagenesis.^a^ | |
| --- | --- |
| *Protein Variant* | Primer nucleotide sequence |
| OVCA2 stop codon | 5’- GGACCAGTTTGCAGAGTCACCAACTTT -3’ |
| OVCA2 S117A | 5’- CGGCCTTCTTGGTTTCGCTCAAGGGGCTGCGCTAGC -3’ |
| OVCA2 D179A | 5’- CCATGTTTTTGGGGACACTGCCAAAGTCATCCCC -3’ |
| OVCA2 H206A | 5’- CCTCACCCACTCTGGTGGCGCCTTCATTCCAGC -3’ |
| ^a^Primers for mutagenesis are one of the two complementary primers used in the mutagenesis reaction. Mutagenic PCR reactions were subjected to the following thermal cycle using a BIO-RAD MyCycler™: 1) initial denaturation at 95°C for 30 s, 2) denaturation at 95°C for 30 s, 3) annealing at temperatures between 52 – 60°C for 60 s, 4) extension at 68°C for 14 min. Steps 2-4 were repeated 18 times. | |
