# Supplementary material for: Comparative analysis of the human serine hydrolase OVCA2 to the model serine hydrolase homolog FSH1 from S. cerevisiae
Source: PLoS One. 2020 Mar 17;15(3):e0230166. doi: 10.1371/journal.pone.0230166 (PMC7077851; doi:10.1371/journal.pone.0230166)
Supplement: S1 Fig — An SDS–PAGE gel (4–20%) showing the protein purification of FSH1. Representative purification samples (10, 25, and 50 mM imidazole washes) shown. FSH1 was purified according to the procedure outlined in Experimental Procedures. The expected molecular weight of FSH1 is 27.3 kDa. The molecular weight was confirmed by comparison to the Kaleidoscope prestained protein standard (Bio-rad laboratories). (DOCX) [file pone.0230166.s007.docx]

**S1 Figure: Purification of FSH1.** An SDS–PAGE gel (4-20%) showing the protein purification of FSH1. Representative purification samples (10, 25, and 50 mM imidazole washes) shown. FSH1 was purified according to the procedure outlined in Experimental Procedures. The expected molecular weight of FSH1 is 27.3 kDa. The molecular weight was confirmed by comparison to the Kaleidoscope prestained protein standard (Bio-rad laboratories).
