# Supplementary material for: Comparative analysis of the human serine hydrolase OVCA2 to the model serine hydrolase homolog FSH1 from S. cerevisiae
Source: PLoS One. 2020 Mar 17;15(3):e0230166. doi: 10.1371/journal.pone.0230166 (PMC7077851; doi:10.1371/journal.pone.0230166)
Supplement: S2 Fig — The folded to unfolded transition for FSH1 (0.3 mg/mL in PBS) was observed by DSF. The measurement was completed in triplicate and is shown ± SD. The majority of the error is smaller than the size of the data marker. (DOCX) [file pone.0230166.s008.docx]

**S2 Figure: Thermal stability and folding of FSH1.** The folded to unfolded transition for FSH1 (0.3 mg/mL in PBS) was observed by DSF. The measurement was completed in triplicate and is shown ± SD. The majority of the error is smaller than the size of the data marker.
