# Supplementary material for: Comparative analysis of the human serine hydrolase OVCA2 to the model serine hydrolase homolog FSH1 from S. cerevisiae
Source: PLoS One. 2020 Mar 17;15(3):e0230166. doi: 10.1371/journal.pone.0230166 (PMC7077851; doi:10.1371/journal.pone.0230166)
Supplement: S3 Fig — Kinetic activity of active site OVCA2 variants (S117A closed circles, D179A open diamonds, and H206A closed squares) compared to the background hydrolysis rate of the same four chromogenic substrates in PBS measured identically. Comparative kinetic activity against a) p-nitrophenyl octanoate (C8), b) p-nitrophenyl decanoate (C10), c) p-nitrophenyl laurate (C12), and d) p-nitrophenyl myristate (C14). Data points were fitted to the Michaelis-Menten equation and are shown ± SD. Each of these plots show that the active site variants for S117A and H206A have activity below the background hydrolysis rate, confirming that substitution of these residues with alanine completely inactivates OVCA2. The D179A variant however shows residual catalytic activity above background hydrolysis rates. (DOCX) [file pone.0230166.s009.docx]

**S3 Figure: Hydrolysis reactions of OVCA2 variants versus background hydrolysis rates.** Kinetic activity of active site OVCA2 variants (S117A closed circles, D179A open diamonds, and H206A closed squares) compared to the background hydrolysis rate of the same four chromogenic substrates in PBS measured identically. Comparative kinetic activity against a) *p-*nitrophenyl octanoate (C8), b) *p-*nitrophenyl decanoate (C10), c) *p-*nitrophenyl laurate (C12), and d) *p-*nitrophenyl myristate (C14). Data points were fitted to the Michaelis-Menten equation and are shown ± SD. Each of these plots show that the active site variants for S117A and H206A have activity below the background hydrolysis rate, confirming that substitution of these residues with alanine completely inactivates OVCA2. The D179A variant however shows residual catalytic activity above background hydrolysis rates.
