# Supplementary material for: Comparative analysis of the human serine hydrolase OVCA2 to the model serine hydrolase homolog FSH1 from S. cerevisiae
Source: PLoS One. 2020 Mar 17;15(3):e0230166. doi: 10.1371/journal.pone.0230166 (PMC7077851; doi:10.1371/journal.pone.0230166)
Supplement: S4 Fig — (A and B) Comparative alignment of FSH1 (brown; PDB ID: 1YCD) to its closest human structural homolog in the PDB (teal; PDB ID: 3U0V), human LYPLAL1 (p-value = 9.8x10^-9^; RMSD = 3.83 Å). Cartoon representation in A with the surface of FSH1 shown in B. (C and D) Comparative alignment of FSH1 (brown; PDB ID: 1YCD) to the second closest human structural homolog in the PDB (grey; PDB ID: 1FJ2), human APT1 (p-value = 2.5x10^-8^; RMSD = 3.13 Å). Cartoon representation in C with the surface of FSH1 shown in D. (E) Close up view of the FSH1 and LYPLAL1 active site surface. The covalent ligand bound to the active site of FSH1 is shown in yellow sticks. The overlap of the LYPLAL1 teal surface with the FSH1 bound ligand shows that the binding surface of LYPLAL1 is shallower than FSH1. (F) Close up view of the APT1 surface (grey; PDB ID: 5SYM) with FSH1 shown in cartoon. The long, open hydrophobic binding pocket of APT1 is shown by the bound ML348 inhibitor, which differs from the closed off pocket of FSH1 (B). All structures aligned using the RCSB pre-calculated alignments.[63] (DOCX) [file pone.0230166.s010.docx]

**S4 Figure: Structural alignment of FSH1 and human APTs.** A and B) Comparative alignment of FSH1 (brown; PDB ID: 1YCD) to its closest human structural homolog in the PDB (teal; PDB ID: 3U0V), human LYPLAL1 (p-value = 9.8x10^-9^; RMSD = 3.83 Å). Cartoon representation in A with the surface of FSH1 shown in B. C and D) Comparative alignment of FSH1 (brown; PDB ID: 1YCD) to the second closest human structural homolog in the PDB (grey; PDB ID: 1FJ2), human APT1 (p-value = 2.5x10^-8^; RMSD = 3.13 Å). Cartoon representation in C with the surface of FSH1 shown in D. E) Close up view of the FSH1 and LYPLAL1 active site surface. The covalent ligand bound to the active site of FSH1 is shown in yellow sticks. The overlap of the LYPLAL1 teal surface with the FSH1 bound ligand shows that the binding surface of LYPLAL1 is shallower than FSH1. F) Close up view of the APT1 surface (grey; PDB ID: 5SYM) with FSH1 shown in cartoon. The long, open hydrophobic binding pocket of APT1 is shown by the bound ML348 inhibitor, which differs from the closed off pocket of FSH1 (B). All structures aligned using the RCSB pre-calculated alignments.
