# Supplementary material for: Comparative analysis of the human serine hydrolase OVCA2 to the model serine hydrolase homolog FSH1 from S. cerevisiae
Source: PLoS One. 2020 Mar 17;15(3):e0230166. doi: 10.1371/journal.pone.0230166 (PMC7077851; doi:10.1371/journal.pone.0230166)
Supplement: Supplementary file 9 [file pone.0230166.s001.docx]

Table S1: Multiple Sequence alignment of OVCA2.[a] Catalytic triad amino acids are shown in bold with grey highlighting.

S. cerevisiae FSH1 (NP_011915.1)	-------------------------MTVQIPKLLFLHGFLQNGKVFSEKSSGIRKLLKKA
S. cerevisiae FSH3 (NP_014923.1)	--------------------------MSEKKKVLMLHGFVQSDKIFSAKTGGLRKNLKKL
S. cerevisiae FSH2 (NP_013949.1)	----------------------------MTKNVLMLHGLAQSGDYFASKTKGFRAEMEKL
S. pombe (NP_588353.1)	--------------------------MSKPLKVLCLHGWIQSGPVFSKKMGSVQKYLSKY
D. melanogaster (NP_650895.1)	MTNNDAAVEAAGSSRASSSKQQLKLEITEKVRVLCLHGYRQNGEAFKNKLGSFRKFANKY
C. elegans (NP_502376.1)	------------------------MSSQPKLRILCLHGYRQCDQSFRQKTGSTRKLVKSL
D. rerio (NP_001018391.1)	-------------------------MSAVPLRILCIHGYRQNGNSFREKTGALRKLLKKQ
X. laevis (XP_018104262.1)	----------------MAATDASRAAQKRVLRVLALHGYRQNERSFWERTGALRKVLRGR
M. musculus (NP_081412.1)	------------------------MAARQTLRVLCLAGFRQSERGFREKTGALRKTLRGR
H. sapiens (NP_543012.1)	------------------------MAAQRPLRVLCLAGFRQSERGFREKTGALRKALRGR
M. fascicularis (XP_005582523.1)	------------------------MAAQRPLRVLCLAGFRQSERGFREKTGALRKALRAR
S. cerevisiae FSH1 (NP_011915.1)	NVQCDYIDAPVLLEKKDLPFEMDDEKWQA------TLDADVNRAWFYHSEI---------
S. cerevisiae FSH3 (NP_014923.1)	GYDLYYPCAPHSIDKKALFQSESEKGRDAAKEFNTSATSDEVYGWFFRNPE--------S
S. cerevisiae FSH2 (NP_013949.1)	GYKLYYPTAPNEFPPADVPDFLGEVIAD----APGDGENTGVLAWLENDP---------S
S. pombe (NP_588353.1)	A-ELHFPTGPVVADEEADPNDEEEKKRLA---ALGGEQNGGKFGWFEVEDF---------
D. melanogaster (NP_650895.1)	A-EFVFITAPHVAKALESA--------------A--EPVPEQRSWWAN-KDDGSFKGTNK
C. elegans (NP_502376.1)	A-EFEFVNGVHSVAVDE--------------------HVDSSRAWWFSNNEAMSFSSRES
D. rerio (NP_001018391.1)	V-ELVFISAPHQVPAIQEENCGTNQQSQT---VS--VGDEDQRGWWFSDVQARSFNAKQD
X. laevis (XP_018104262.1)	A-ELITFSAPLVVPDPDA---EPS--DGD---PN--SSQDESRGWWFSNPQQNSFDAMEE
M. musculus (NP_081412.1)	A-ELVCLSGPHPVPEAAAPEGSCP--DSG---PC--SPEEQPRGWWFSEEEADVFSALEE
H. sapiens (NP_543012.1)	A-ELVCLSGPHPVPDPPGPEGARS--DFG---SC--PPEEQPRGWWFSEQEADVFSALEE
M. fascicularis (XP_005582523.1)	A-ELVCLSGPHPVPDAPGPEGARS--DFG---SC--PPEEQPRGWWFSEQEADVFSALEE
S. cerevisiae FSH1 (NP_011915.1)	-SHELDISEGLKSVVDHIKANGPYDGIVGF**S**QGAALSSIITNKISE-LVPDH-----PQF
S. cerevisiae FSH3 (NP_014923.1)	FNSFQIDQKVFNYLRNYVLENGPFDGVIGF**S**QGAGLGGYLVTDFNR--ILNLTDEQQPAL
S. cerevisiae FSH2 (NP_013949.1)	TGGYFIPQTTIDYLHNYVLENGPFAGIVGF**S**QGAGVAGYLATDFNG--LLGLTTEEQPPL
S. pombe (NP_588353.1)	KNTYGSWDESLECINQYMQEKGPFDGLIGF**S**QGAGIGAMLAQMLQP-GQPPNPYVQHPPF
D. melanogaster (NP_650895.1)	GGPAFGFQESLRCVEEAWRTQGPFQGLLGF**S**QGACFVGLICGLAKKKLTS-------IRP
C. elegans (NP_502376.1)	TEVAVGFEESVAAVVKFIEENGPFDGLLGF**S**QGASMVHLLIAKAQL-GEIKL-----PGI
D. rerio (NP_001018391.1)	CESSLGLEESIEAVKAALKDLGPFSGILGF**S**QGAALVAMLCALQEQKLEPDF-----NF-
X. laevis (XP_018104262.1)	SNTCSGLEASLDTVAKAFSELGPFDGILGF**S**QGAALVAIICALKQH-GDPRF-----HFN
M. musculus (NP_081412.1)	STVCRGLQEALETVARALDTLGPFDGLLGF**S**QGAALAAYVCALGQA-GDPRF-----PLP
H. sapiens (NP_543012.1)	PAVCRGLEESLGMVAQALNRLGPFDGLLGF**S**QGAALAALVCALGQA-GDPRF-----PLP
M. fascicularis (XP_005582523.1)	PAVCRGLEESLGMVAQALSRLGPFDGLLGF**S**QGAALAAFICALGQA-GDPRF-----PLP
S. cerevisiae FSH1 (NP_011915.1)	KVSVVISGYSFTEPDPEHPGELRITEKFRDSFAVKPDMKTKMIFIYGAS**D**QAVPSVRSKY
S. cerevisiae FSH3 (NP_014923.1)	KFFISFSGFKLEDQSYQKE-------------YH-RIIQVPSLHVRGEL**D**EVVAESRIMA
S. cerevisiae FSH2 (NP_013949.1)	EFFMAVSGFRFQPQQYQEQ-------------YDLHPISVPSLHVQGEL**D**TITEPAKVQG
S. pombe (NP_588353.1)	KFVVFVGGFRAEKPEFDH--------------FYNPKLTTPSLHIAGTS**D**TLVPLARSKQ
D. melanogaster (NP_650895.1)	EFAVLASGFLSGSLVHMS--------------AYEEAISIPTLHIYGQT**D**EIIPKEMSES
C. elegans (NP_502376.1)	RFAIFFSGFLSLSSKHDSL-------------TLLRIKEFPSMHVFGDA**D**EIVARPKSEK
D. rerio (NP_001018391.1)	RFAILVAGFRSACLEHQRF-------------YEGPVITIPSLHVFGQD**D**RVIPEEMSRD
X. laevis (XP_018104262.1)	-FAILVAGFKSRSSEH-TK-------------HYQQPNTVPSLHVIGET**D**RVISADMSRE
M. musculus (NP_081412.1)	RFIILVSGFCPRG--LKEP-------------ILQSPMSLPSLHVFGDT**D**RVIPSQESMQ
H. sapiens (NP_543012.1)	RFILLVSGFCPRGIGFKES-------------ILQRPLSLPSLHVFGDT**D**KVIPSQESVQ
M. fascicularis (XP_005582523.1)	RFIILVSGFCPRGLGFKES-------------ILQRPLSLPSLHVFGDT**D**KVIPSPESMQ
S. cerevisiae FSH1 (NP_011915.1)	LYDIYLKAQNGNKEKVLAYEHPGG**H**MVPNKKDIIRPIVEQITSSLQEASE----------
S. cerevisiae FSH3 (NP_014923.1)	LYESWPD------NKRTLLVHPGA**H**FVPNSKPFVSQVCNWIQGITSKEGQEHNAQPEVD-
S. cerevisiae FSH2 (NP_013949.1)	LYNSCTE------DSRTLLMHSGG**H**FVPNSRGFVRKVAQWLQQLT---------------
S. pombe (NP_588353.1)	LVERCE-------NA-HVLLHPGQ**H**IVPQQAVYKTGIRDFMFSAPTKEPTKHPRDLTMIV
D. melanogaster (NP_650895.1)	LAARFK-------NA-EVLEHSGG**H**YFPATAQQKQTFINFFQDRLQEYLEHEE--LQQSG
C. elegans (NP_502376.1)	MADMFDV------EP-LRIAHDGG**H**VVPSMSKHKEKIAGFMREQLDRKIENN--------
D. rerio (NP_001018391.1)	LLPAFD-------GA-QVLLHPGG**H**FVPAASSHRQTYQDFLKRFQ---------------
X. laevis (XP_018104262.1)	LVSHFE-------NP-FIVMHPGG**H**YVPACAPQKKVYWEFLDKFLS--------------
M. musculus (NP_081412.1)	LASRFL-------GA-VTLTHSGG**H**FIPAAASQRQAYLKFLDQFAE--------------
H. sapiens (NP_543012.1)	LASQFP-------GA-ITLTHSGG**H**FIPAAAPQRQAYLKFLDQFAE--------------
M. fascicularis (XP_005582523.1)	LASRFP-------GA-ITLTHSGG**H**FIPAAAAQRQAYLKFLDQFAE--------------
S. cerevisiae FSH1 (NP_011915.1)	--
S. cerevisiae FSH3 (NP_014923.1)	RKQFDKPQLEDD----------L------LDMIDSLGKL---------------------
S. cerevisiae FSH2 (NP_013949.1)	--
S. pombe (NP_588353.1)	AVSSPNLGIGKKNSMPWHIKQEMAYFANVTSSTESSGQLEEGKSKIMNVVIMGRSCYDSL
D. melanogaster (NP_650895.1)	NASFVDSGAEDDNDA------EV---AAMT------AELDESD-----------------
C. elegans (NP_502376.1)	--
D. rerio (NP_001018391.1)	--
X. laevis (XP_018104262.1)	--
M. musculus (NP_081412.1)	--
H. sapiens (NP_543012.1)	--
M. fascicularis (XP_005582523.1)	--

[a] The amino acid sequence of OVCA2 was aligned using Clustal Omega. Sequences for alignment were chosen based on protein BLAST analysis of OVCA2 and extracting unique protein sequences with significant percent similarity (> 20%).

[b] The amino acid sequence of the homolog from *S. pombe* was shortened by 163 amino acids as this section did not align with any other homologues.
