# Supplementary material for: Comparative analysis of the human serine hydrolase OVCA2 to the model serine hydrolase homolog FSH1 from S. cerevisiae
Source: PLoS One. 2020 Mar 17;15(3):e0230166. doi: 10.1371/journal.pone.0230166 (PMC7077851; doi:10.1371/journal.pone.0230166)
Supplement: Supplementary file 10 [file pone.0230166.s005.docx]

Table S5: Multiple sequence alignment of OVCA2, FSH1, and human APTs. Catalytic triad amino acids are shown in bold with grey highlighting.

H. sapiens LYPLAL1 (NP_620149.2)	MAAA---SGSVLQRCIVSPAGRHSASLIFLHGSGDSGQGLRMWIKQVLNQDLTFQHIKII
H. sapiens APT1 (NP_006321.1)	MCGNNMSTPLPA---IVPAARKATAAVIFLHGLGDTGHGWAEAF-----AGIRSSHIKYI
H. sapiens APT2 (NP_009191.1)	MCGNTMSVPLLTDAATVSGAERETAAVIFLHGLGDTGHSWADAL-----STIRLPHVKYI
S. cerevisiae FSH1 (NP_011915.1)	------------------MTV-QIPKLLFLHGFLQNGKVFSEKSSGIRKLLKKA-NVQCD
H. sapiens OVCA2 (NP_543012.1)	------------------MAAQRPLRVLCLAGFRQSERGFREKTGALRKALRGRAELVCL
H. sapiens LYPLAL1 (NP_620149.2)	YPTAPPR------SYTPM--------KGGISNVWFDRFKITND---------CPEHLESI
H. sapiens APT1 (NP_006321.1)	CPHAPVR------PVTLN--------MNVAMPSWFDIIGLSPD---------SQEDESGI
H. sapiens APT2 (NP_009191.1)	CPHAPRI------PVTLN--------MKMVMPSWFDLMGLSPD---------APEDEAGI
S. cerevisiae FSH1 (NP_011915.1)	YIDAPVLLEKKDLPFEMDDEKWQATLDADVNRAWFYHSEISHE----------LDISEGL
H. sapiens OVCA2 (NP_543012.1)	SGPHPVP--DPPGP-EGARSDFGSCPPEEQPRGWWFSEQEADVFSALEEPAVCRGLEESL
H. sapiens LYPLAL1 (NP_620149.2)	DVMCQVLTDLIDEEVKSGIKKNRILIGGF**S**MGGCMAIHLAYRNH-----QDVAGVF-ALS
H. sapiens APT1 (NP_006321.1)	KQAAENIKALIDQEVKNGIPSNRIILGGF**S**QGGALSLYTALTTQ-----QKLAGVT-ALS
H. sapiens APT2 (NP_009191.1)	KKAAENIKALIEHEMKNGIPANRIVLGGF**S**QGGALSLYTALTCP-----HPLAGIV-ALS
S. cerevisiae FSH1 (NP_011915.1)	KSVVDHIKA-------NGP-Y--DGIVGL**S**QGAALSSIITNKISELVPDHPQFKVSVVIS
H. sapiens OVCA2 (NP_543012.1)	GMVAQALNR-------LGP-F--DGLLGF**S**QGAALAALVCALGQAGDPRFPLPRFILLVS
H. sapiens LYPLAL1 (NP_620149.2)	SFLNKA--SA----------VYQALQKSNGVLPELFQCHGTA**D**ELVLHSWAEETNSMLKS
H. sapiens APT1 (NP_006321.1)	CWLPLR--AS----------FPQGPIGGANRDISILQCHGDC**D**PLVPLMFGSLTVEKLKT
H. sapiens APT2 (NP_009191.1)	CWLPLH--RA----------FPQAAN-GSAKDLAILQCHGEL**D**PMVPVRFGALTAEKLRS
S. cerevisiae FSH1 (NP_011915.1)	GYSFTEPDPEHPGELRITEKFRDSFAVKPDMKTKMIFIYGAS**D**QAVPSVRSKYLYDIYLK
H. sapiens OVCA2 (NP_543012.1)	GFCPRGI------------GFKESILQR-PLSLPSLHVFGDT**D**KVIPSQESVQLASQF--
H. sapiens LYPLAL1 (NP_620149.2)	LG-----VTTKFHSFPNVY**H**ELSKTELDILKLWILTKLPGEMEKQK
H. sapiens APT1 (NP_006321.1)	LVN---PANVTFKTYEGMM**H**SSCQQEMMDVKQFIDKLLPPID----
H. sapiens APT2 (NP_009191.1)	VVT---PARVQFKTYPGVM**H**SSCPQEMAAVKEFLEKLLPPV-----
S. cerevisiae FSH1 (NP_011915.1)	AQNGNKEKVLAYEHPGG**H**MVPNKKDIIRPIVEQITSSLQEASE---
H. sapiens OVCA2 (NP_543012.1)	------PGAITLTHSGG**H**FIPAAAPQRQAYLKFLDQ-FAE------
